# Neural connectivity in Internet gaming disorder and alcohol use disorder: A resting-state EEG coherence study

**DOI:** 10.1038/s41598-017-01419-7

**Published:** 2017-05-02

**Authors:** Su Mi Park, Ji Yoon Lee, Yeon Jin Kim, Jun-Young Lee, Hee Yeon Jung, Bo Kyung Sohn, Dai Jin Kim, Jung-Seok Choi

**Affiliations:** 1 Department of Psychiatry, SMG-SNU Boramae Medical Center, Seoul, Republic of Korea; 2 Department of Clinical Medical Sciences, Seoul National University College of Medicine, Seoul, Republic of Korea; 3 Interdisciplinary Program in Neuroscience, Seoul National University College of Natural Sciences, Seoul, Republic of Korea; 4 Department of Psychiatry and Behavioral Science, Seoul National University College of Medicine, Seoul, Republic of Korea; 5 Department of Psychiatry, Seoul St. Mary’s Hospital, The Catholic University of Korea College of Medicine, Seoul, Republic of Korea

## Abstract

The present study compared neural connectivity and the level of phasic synchronization between neural populations in patients with Internet gaming disorder (IGD), patients with alcohol use disorder (AUD), and healthy controls (HCs) using resting-state electroencephalography (EEG) coherence analyses. For this study, 92 adult males were categorized into three groups: IGD (n = 30), AUD (n = 30), and HC (n = 32). The IGD group exhibited increased intrahemispheric gamma (30–40 Hz) coherence compared to the AUD and HC groups regardless of psychological features (e.g., depression, anxiety, and impulsivity) and right fronto-central gamma coherence positively predicted the scores of the Internet addiction test in all groups. In contrast, the AUD group showed marginal tendency of increased intrahemispheric theta (4–8 Hz) coherence relative to the HC group and this was dependent on the psychological features. The present findings indicate that patients with IGD and AUD exhibit different neurophysiological patterns of brain connectivity and that an increase in the fast phasic synchrony of gamma coherence might be a core neurophysiological feature of IGD.

## Introduction

Internet gaming disorder (IGD) is the repetitive use of Internet-based games that leads to significant difficulties in social and psychological functioning^1^. The concepts underlying IGD differ from those associated with general Internet addiction but, because the terminology of IGD is relatively new, it has been commonly referred to as Internet use disorder or Internet addiction for the past several decades^2^. Thus, even though the present study focused on IGD, evidence regarding Internet addiction will also be presented as an analogy and to support the present findings.

IGD is categorized as a behavioral addiction similar to pathological gambling, sexual activity, exercise, and shopping^3^ because these diverse behavioral addiction disorders share a number of clinical features including repetitive behavior despite negative results, a lack of control over urges, and cravings prior to engagement in the behavior^4^. According to a review by Olsen, non-drug addictions alter neuroplasticity in brain areas associated with reward processing and drug addiction^5^. The inclusion of behavioral addictions as a new class of disorders was proposed for the Diagnostic and Statistical Manual of Mental Disorders, Fifth Edition (DSM-5), but only Gambling Disorder (GD) was categorized as such^6^. However, it is suggested in the appendix of the DSM-5 that IGD be further assessed as an addictive condition because it has been associated with depressed mood, anxiety, and aggressiveness as well as with psychiatric disorders such as attention deficit hyperactivity disorder (ADHD) and obsessive–compulsive disorder (OCD)^7–12^. Thus, there is a pressing need to elucidate further the neurobiological pathophysiology underlying IGD to better understand this disorder.

Electroencephalographic (EEG) measurements of brain wave coherence may be utilized to infer abnormalities in the functional organization of the brain^13, 14^. EEG coherence reflects brain dynamics in terms of the coupling and functional association of two brain regions^15, 16^ and is primarily a measure of phasic correlation. This measure implies functional cortical connectivity either directly via the corticocortical fiber systems or indirectly through networks that include other cortical and/or subcortical structures^17, 18^. High coherence between two EEG signals may present as synchronized neuronal oscillations, which suggest the functional integration of two neural populations, whereas low coherence represents two independently active populations and is indicative of functional segregation^19^. EEG coherence during the resting state is associated with functional magnetic resonance imaging (fMRI) measures of functioning in the default mode network (DMN) and is thought to be useful for characterizing abnormalities in patients with psychiatric disorders such as schizophrenia, bipolar disorder, and obsessive compulsive disorder (OCD)^20–23^. While fMRI studies are useful for the characterization of the spatial distribution of resting-state networks, EEG measures provide unique information about the synchronization of neuronal activity at high temporal frequencies.

Several studies have shown that individuals with a substance-use disorder (SUD) exhibit abnormalities in EEG coherence. For example, individuals with an alcohol-use disorder (AUD) exhibit increased interhemispheric coherence relative to unaffected individuals^24, 25^ and patients with long-term abstinent and non-abstinent AUD show significant increases in bilateral, intrahemispheric, and posterior coherence values in the alpha and beta frequency bands^26^. These findings suggest that increased EEG coherence may serve as an endophenotype for AUD. Similarly, Dafters *et al*. observed reduced coherence in the posterior brain regions of 3,4-methylenedioxymethamphetamine (MDMA) users^27^. In terms of behavioral addictions, one study found an association between neural connectivity and compulsive buying behavior using EEG coherence analyses^28^, which suggests that dysfunctional cortical connectivity may also be associated with addictive behaviors.

Although few studies have used EEG to investigate brain connectivity in IGD patients, a recent study found that adolescents with an Internet addiction exhibit increased gamma coherence compared to healthy control (HC) subjects^29^. Furthermore, resting-state fMRI studies have consistently identified dysfunctional connectivity in IGD patients^30^. For example, Wang *et al*. observed altered interhemispheric resting-state functional connectivity in the bilateral prefrontal lobe in individuals with IGD^31^. A comparison of intrinsic local connectivity among IGD patients, AUD patients, and HCs using fMRI revealed significant increases in regional homogeneity (ReHo) in the posterior cingulate cortex (PCC) of the IGD and AUD groups as well as decreases in ReHo in the right superior temporal gyrus (STG) of IGD patients compared to AUD patients and HCs^32^.

Thus, the present study aimed to investigate neuronal connectivity in patients with IGD and to clarify the neurophysiological features of this population by comparing their EEG data with those of AUD patients and HCs. AUD was selected as a traditional SUD because disruptions in the functional brain networks of AUD patients have been repeatedly examined^33–35^. Previous studies have suggested that IGD is a behavioral addiction that has similarities and differences with AUD, which is a depressant SUD^36, 37^. However, to the best of our knowledge, no direct comparisons of EEG coherence in IGD and AUD patients have been conducted to date.

A primary aim of the present study was to detail similarities and differences between IGD and AUD patients; it was hypothesized that the IGD patients and AUD patients would exhibit abnormal increases in EEG coherence patterns relative to HCs. Questionnaires were used to determine the psychological characteristics of the participants and the intra- and interhemispheric EEG coherence values were assessed as the primary variables of interest in this study. Additionally, the relationships between EEG features and the degree of addiction tendency for Internet gaming were investigated.

## Results

### Demographic and psychological variables

See Table 1 for the detailed results of the inter-group comparisons of the demographic and psychological characteristics.

**Table 1:** Demographic and psychological characteristics of the IGD, AUD, and HC groups.

	*IGD* (*n* = *30*)	*AUD* (*n* = *30*)	*HC* (*n* = *32*)	*F*	*P*	*Post hoc*
	Mean (S.D.)					
Age	23.267 (5.152)	29.867 (7.133)	24.969 (3.703)	11.777	<0.001	A > I, A > H
Education	12.931 (1.831)	14.143 (2.663)	14.612 (2.390)	4.153	0.019	
IQ	113.833 (13.217)	110.689 (12.559)	119.531 (9.808)	4.357	0.016	H > A
IAT	69.267 (14.776)	30.846 (10.364)	29.290 (8.533)	113.758	<0.001	I > A, I > H
AUDIT	6.172 (4.465)	24.071 (5.422)	5.226 (3.676)	156.429	<0.001	A > I, A > H
***controlling age, education, and IQ covariates***						
BDI-II	18.448 (10.270)	24.321 (16.764)	3.710 (3.892)	20.347	<0.001	I > H, A > H
BAI	15.241 (13.144)	23.143 (16.770)	5.806 (5.431)	12.517	<0.001	A > I, A > H
BIS-11	68.690 (13.148)	72.643 (13.276)	55.226 (8.865)	18.080	<0.001	I > H, A > H

Group differences for demographic and psychological variables were analyzed. Bonferroni correction was applied to the post hoc analyses, and the significance level was set at 0.0167. IGD (I) = Internet gaming disorder; AUD (A) = alcohol use disorder; HC (H) = healthy control; IQ = intelligence quotient; IAT = Young’s Internet Addiction Test; AUDIT = Alcohol Use Disorder Identification Test; BDI-II = Beck Depression Inventory-II; BAI = Beck Anxiety Inventory; and BIS-11 = Barratt Impulsivity Scale-11.

### EEG Coherence

- **Coherence analyses without controlling for the effects of the psychological characteristics**

**Figure 1:**
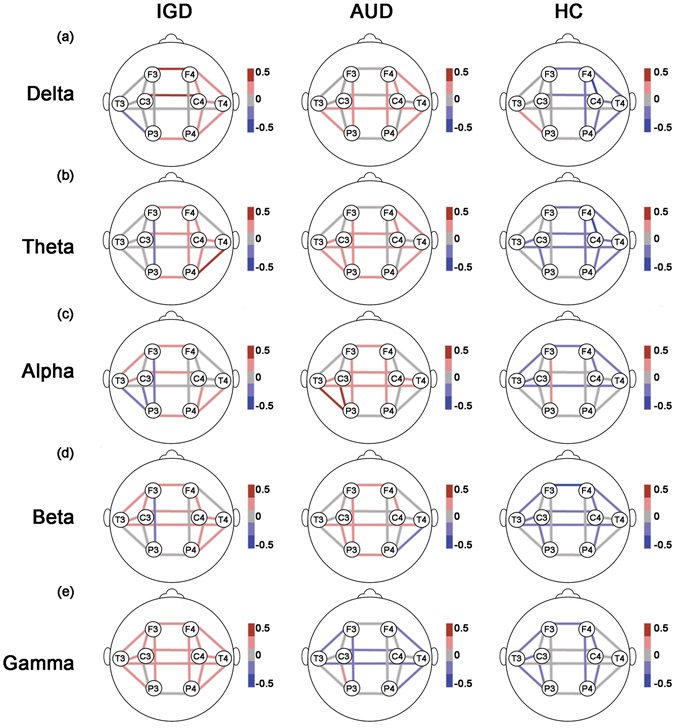
Coherence without controlling effects of psychological covariates. The lines represent estimated coherence means of the groups by GEE at each location for the (**a**) delta, (**b**) theta, (**c**) alpha, (**d**) beta, and (**e**) gamma bands. The effects of demographic data (age, education, and IQ) were controlled; however, that of psychological data (scores on the BDI-II, BAI, and BIS-11) were not controlled in analyses. The meaning of bar scales ranging from −0.5 (blue) to 0.5 (red). Fisher’s Z-transformed scores were used for the EEG data. IGD = Internet gaming disorder; AUD = alcohol use disorder; HC = healthy control; GEE = generalized estimating model; EEG = electroencephalography; IQ = intelligence quotient; BDI-II = Beck Depression Inventory-II; BAI = Beck Anxiety Inventory; and BIS-11 = Barratt Impulsivity Scale-11.

Figure 1 presents the overall features of the estimated means (Ms) of coherence at individual EEG electrode pairs in each frequency band for the three groups without controlling for the effects of the psychological characteristics.

- ***Intrahemispheric coherence without controlling for the effects of the psychological characteristics***
  GEE analyses of intrahemispheric coherence while controlling for the effects of the demographic variables but not the effects of the psychological variables revealed significant group effects in the theta (${x}_{(2)}^{2}=9.117$, *P* = 0.010), beta (${x}_{(2)}^{2}=8.414$, *P* = 0.015), and gamma (${x}_{(2)}^{2}=10.826$, *P* = 0.004) bands (Table 2). More specifically, the IGD group (M [S.E.M.] = 0.107 [3.742]) showed greater coherence in the beta band than the HC group (M [S.E.M.] = −0.116 [3.730]; *P = *0.014), and the IGD group (M [S.E.M.] = 0.146 [3.769]) showed greater coherence in the gamma band than the HC group (M [S.E.M.] = −0.104 [3.757]; *P = *0.005). The AUD group {M [standard error of the mean (S.E.M.)] = 0.126 [3.883]} exhibited marginal tendency of greater coherence in the theta band than the HC group (M [S.E.M.] = −0.117 [3.855]; P = 0.020).

**Table 2:** Factor effects on EEG intrahemispheric coherence.

	Without controlling psychological covariates			With controlling psychological covariates		
	*χ* ^*2*^	*p*	*Post hoc*	*χ* ^*2*^	*P*	*Post hoc*
**Delta**						
Group	6.033	0.049		1.568	0.456	
Group × Region	2.795	0.986		3.363	0.972	
Group × Hemisphere	6.771	0.034		6.714	0.035	
Group × Region × Hemisphere	0.964	1.000		1.192	1.000	
**Theta**						
Group	9.117	0.010		1.414	0.493	
Group × Region	2.628	0.989		3.006	0.981	
Group × Hemisphere	5.746	0.057		4.090	0.129	
Group × Region × Hemisphere	2.544	0.990		2.651	0.988	
**Alpha**						
Group	3.047	0.218		6.935	0.031	
Group × Region	4.831	0.902		5.458	0.859	
Group × Hemisphere	1.329	0.515		0.909	0.635	
Group × Region × Hemisphere	5.007	0.891		4.566	0.918	
**Beta**						
Group	8.414	0.015	I > H	10.165	0.006	
Group × Region	4.963	0.894		5.626	0.846	
Group × Hemisphere	0.874	0.646		0.893	0.640	
Group × Region × Hemisphere	5.001	0.891		4.676	0.912	
**Gamma**						
Group	10.826	0.004	I > H	25.737	< 0.001	I > A, I > H
Group × Region	2.211	0.994		2.477	0.991	
Group × Hemisphere	0.467	0.792		0.571	0.752	
Group × Region × Hemisphere	0.032	1.000		1.279	0.999	

**Figure 2:**
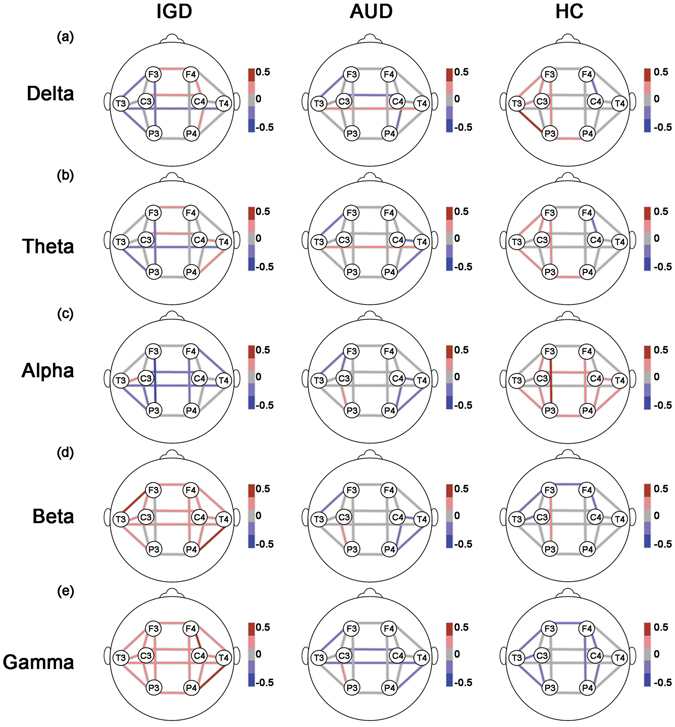
Coherence with controlling effects of psychological covariates. The lines represent estimated coherence means of the groups by GEE at each location for the (**a**) delta, (**b**) theta, (**c**) alpha, (**d**) beta, and (**e**) gamma bands. Both effects of demographic data (age, education, and IQ) and psychological data (scores on the BDI-II, BAI, and BIS-11) were controlled in analyses. The meaning of bar scales ranging from −0.5 (blue) to 0.5 (red). Fisher’s Z-transformed scores were used for the EEG data. IGD = Internet gaming disorder; AUD = alcohol use disorder; HC = healthy control; GEE = generalized estimating model; EEG = electroencephalography; IQ = intelligence quotient; BDI-II = Beck Depression Inventory-II; BAI = Beck Anxiety Inventory; and BIS-11 = Barratt Impulsivity Scale-11.
- ***Intrahemispheric coherence while controlling for the effects of the psychological characteristics***
  GEE analyses of intrahemispheric coherence while controlling for the effects of the demographic and psychological variables revealed significant group effects in the beta (${x}_{(2)}^{2}=10.165$, *P* = 0.006) and gamma (${x}_{(2)}^{2}=25.737$, *P* < 0.001) bands (Table 2). More specifically, the IGD group (M [S.E.M.] = 0.230 [7.995]) showed greater coherence in the gamma band than both the AUD (M [S.E.M] = −0.077 [7.986]; *P* = 0.005) and HC (M [S.E.M.] = −0.088 [8.015]; *P* < 0.001) groups. Also, the IGD group (M [S.E.M.] = 0.135 [7.844]) exhibited marginal tendency of greater coherence in the beta band than the AUD group (M [S.E.M.] = 0.019 [7.835]; *P* = 0.023).
  There were no significant group or group × region effects for the delta, theta, or alpha bands (all *P*-values > 0.0167) and no significant group × region effects for the beta or gamma bands (all *P*-values > 0.0167).
- ***Interhemispheric coherence while controlling for the effects of the psychological characteristics***

Effects on EEG intrahemispheric coherence with or without controlling effects of psychological variables (scores on the BDI-II, BAI, and BIS-11) were analyzed separately for each frequency band. Effects of demographic data (age, education, and IQ) variables were controlled in all conditions. Bonferroni correction was applied to the post hoc analysis, and the significance level was set at 0.0167. Fisher’s Z-transformed scores were used for the EEG data. The group factor consists of IGD (n = 30), AUD (n = 30), and HC (n = 32). The region factor consists of following pairs: fronto-central, fronto-temporal, fronto-parietal, centro-temporal, centro-parietal, temporoparietal. The hemisphere factor consists of left and right side. EEG = electroencephalography; IGD (I) = Internet gaming disorder; AUD (A) = alcohol use disorder; HC (H) = healthy control; IQ = intelligence quotient; BDI-II = Beck Depression Inventory-II; BAI = Beck Anxiety Inventory; and BIS-11 = Barratt Impulsivity Scale-11.
  There were no significant group or group × region effects in the delta or alpha bands (all *P*-values > 0.0167) or significant group × region effects in the theta, beta, or gamma bands (all *P*-values > 0.0167).
- ***Interhemispheric coherence without controlling for the effects of the psychological characteristics***
  Analyses of interhemispheric coherence while controlling for the effects of the demographic variables but not the psychological variables revealed that there were no significant group or group × region effects for the delta, theta, alpha, beta, or gamma bands (all *P*-values > 0.0167).
- **Coherence while controlling for the effects of the psychological characteristics**
  Figure 2 presents overall features of the estimated Ms of coherence at individual EEG electrode pairs in each frequency band for the three groups while controlling for the effects of the psychological variables.

Analyses of interhemispheric coherence while controlling for the effects of the demographic and psychological variables revealed that there were no significant group differences or group × region effects for any of the frequency bands (all *P*-values > 0.0167).

### Relationships among the variables

**Figure 3:**
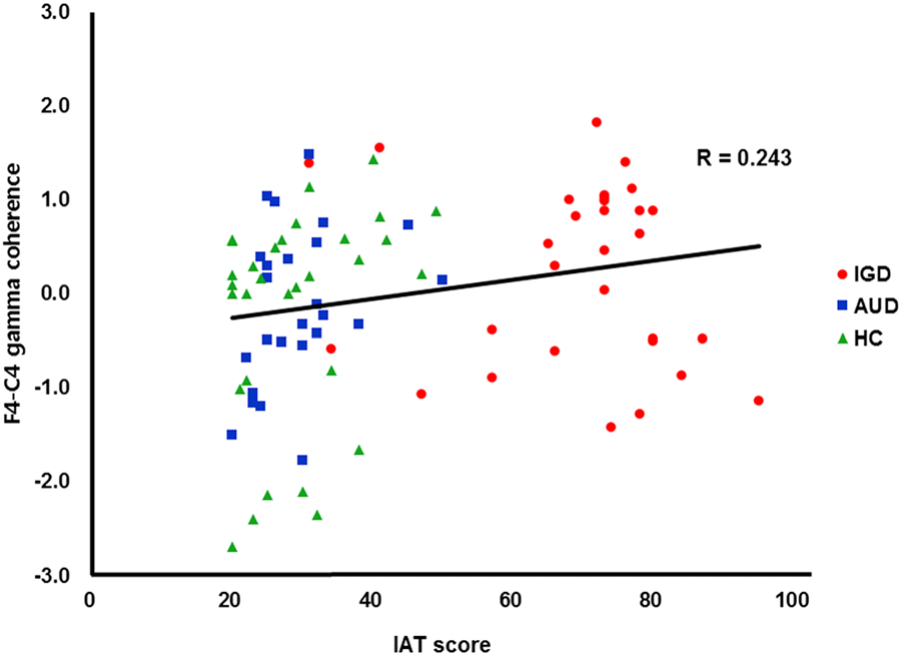
Correlation between the F4-C4 gamma coherence and the score on IAT. The scatter plot presents the correlation between variables in IGD (red circle), AUD (blue square), and HC (green triangle) groups. Fisher’s Z-transformed scores were used for the EEG data. 2000 sample bootstrapping was applied with Pearson’s correlation, and the significance level was set at 0.050. IGD = Internet gaming disorder; AUD = alcohol use disorder; HC = healthy control﻿; EEG = electroencephalography.
  Next, hierarchical multiple regressions were conducted to determine the relationships between intrahemispheric gamma coherence and the IAT scores in all three groups. Several models were constructed to predict the IAT score: Model 1 included only F4-C4 gamma coherence as a dependent variable; Model 2 included the demographic variables (age, education, and IQ) and the factors included in Model 1; and Model 3 included the psychological variables (Beck Depression Inventory-II [BDI-II], Beck Anxiety Inventory [BAI], and Barratt Impulsiveness Scale-11 [BIS-11]) and the factors included in Model 2. The effect of F4-C4 gamma coherence in terms of predicting the IAT score remained significant after the demographic variables were input (*B* = 5.268, *P* = 0.017 in Model 1 and *B* = 5.055, *P* = 0.025 in Model 2; Table 3) and age was a significant factor for predicting IAT scores in Model 2 (*B* = −1.011, *P* = 0.017). However, Model 3 did not significantly change from Model 2 (^*∆*^ *P* > 0.050).

- **EEG coherence and IGD**
  Based on the significant group differences revealed by the GEE analyses, the correlations between the beta and gamma intrahemispheric coherence values of each electrode pair and the degree of Internet addiction tendency, as assessed by the Internet Addiction Test (IAT), were investigated. For all groups, there was a significant correlation between F4-C4 gamma coherence and the IAT score (*R* = 0.243, *P* = 0.012; Fig. 3) but the correlations between the EEG variables and the severity of IGD were not significant in the IGD group (all *P-*values > 0.050). The intrahemispheric beta coherence values were not correlated with the IAT score.

**Table 3:** Hierarchical regressions predicting the score on IAT in the IGD, AUD, and HC groups.

Model	DV	^*∆*^ *R* ^*2*^	^*∆*^ *F*	^*∆*^ *P*	*B*	*P*
**1**		0.058	5.099	0.027		
	F4-C4 gamma coherence				0.241	0.012
**2**		0.118	3.318	0.013		
	F4-C4 gamma coherence				0.231	0.025
	Age				−0.283	0.017
	Education				−0.141	0.127
	IQ				0.097	0.380
**3**		0.077	2.267	0.054		
	F4-C4 gamma coherence				0.221	0.029
	Age				−0.310	0.014
	Education				−0.075	0.448
	IQ				0.193	0.127
	BDI-II				0.248	0.301
	BAI				−0.140	0.495
	BIS-11				0.181	0.226

Hierarchical regression models predicting the score on IAT were conducted for all participants in this study. Fisher’s Z-transformed scores were used for the EEG data. 2000 sample bootstrapping was applied with regression analyses, and the significance level was set at 0.050. IAT = Young’s Internet Addiction Test; IGD = Internet gaming disorder; AUD = alcohol use disorder; HC = healthy control; F3-C3 = left hemispheric frontocontral, IQ = intelligence quotient; BDI-II = Beck Depression Inventory-II; BAI = Beck Anxiety Inventory; and BIS-11 = Barratt Impulsivity Scale-11; EEG = electroencephalography.
- **EEG coherence and AUD**

AUD severity was assessed using the Alcohol Use Disorders Identification Test (AUDIT) and, based on the GEE results, the correlation of the AUDIT score with the interhemispheric theta coherence value was evaluated. For all groups, there were no significant correlations between the EEG variables and the AUDIT scores (all *P-*values > 0.050) but there was a significant correlation between T3-P3 theta coherence and the AUDIT score in the AUD group (*R* = 0.424, *P* = 0.014). A regression analysis revealed that T3-P3 theta coherence positively predicted the AUDIT scores in the AUD group (*B* = 2.320, *P* = 0.036) but an additional model that also included the demographic and psychological variables did not reach significance (^*∆*^ *P* > 0.050).

## Discussion

The primary goal of the present study was to identify the electrophysiological features of IGD using resting-state EEG coherence by comparing the EEG data of IGD patients with those of AUD patients and HCs. IGD subjects exhibited increased intrahemispheric gamma coherence irrespective of the psychological variables (depression, anxiety, and impulsivity) while AUD subjects showed increased intrahemispheric theta coherence that was dependent on the psychological variables. Additionally, right fronto-central gamma coherence positively predicted the scores of IAT. Thus, the present results suggest that IGD and AUD are associated with different neuropathological patterns of brain connectivity and that heightened neural synchrony in the fast-frequency bands may be a core neuropathological feature of IGD.

The present findings are consistent with those of Kwan and Choi who reported that adolescents with an Internet addiction exhibit increased resting-state gamma coherence^29^. The mechanisms underlying gamma oscillations are associated with high levels of alertness and arousal whereas slow-wave oscillations are associated with a low level of alertness^38^. Similarly, abnormal gamma oscillations are related to dysfunctional activity in the dopaminergic system in patients with psychiatric disorders, GABAergic disinhibition, excitatory activation of the brain, and seeking behaviors related to addiction^39–44^. Interestingly, gamma phasic synchrony plays a role in the learning process under the loss condition in a monetary reward task but this change is modulated by impulsivity^45^. This finding implies that increased gamma coherence is associated with inhibition and punishment-related learning in HCs but has another meaning in individuals with impulsivity problems; i.e., it may be associated with increased risk-taking behavior. Although the findings of that study regarding gamma coherence were related to task-specific conditions, abnormal gamma activity has been identified in resting-state EEG studies as well. For example, Choi *et al*. found that individuals with an Internet addiction show decreased beta and increased gamma absolute power values^46^, which indicates that the functionality of the DMN may be altered in patients with Internet addictions.

Accordingly, several fMRI studies have shown that IGD patients exhibit dysfunction within reward circuits. For example, Ding *et al*. found that adolescents with IGD show increased PCC connectivity relative to HCs and that this PCC connectivity is correlated with the severity of IGD symptoms^47^. Similarly, individuals with IGD exhibit greater brain activation in reward processing-related regions, such as the dorsolateral prefrontal cortex, PCC, anterior cingulate cortex, and precuneus, in response to gaming cues compared to HCs^48^. In the present study, gamma coherence in the fronto-central area predicted the IAT scores in all groups. Taken together, the present findings concerning increased gamma coherence in IGD patients during the resting-state indicate that their behavioral changes may be related to an abnormal excitatory system and dysfunctional reward-sensitive responses. Future EEG studies using source localization and/or an integrative approach in conjunction with other methods are needed to clarify the roles of gamma synchrony and the DMN in IGD.

It is also plausible that heightened coherence in the fast-frequency EEG bands of IGD patients reflects a state of hyperarousal within the visual system. Crick and Koch proposed that neural synchrony in the gamma frequency band may be a neural correlate of visual awareness^49^. Accordingly, IGD patients spend large amounts of time playing online games and are overly exposed to visual stimuli, which could change the default mode of the neural network to a restless state even during a resting state with the eyes closed. Emotional states such as depression and anxiety may also be related to arousal levels and EEG fast-frequency band activities^50^. However, this explanation is rather unlikely because, in the present study, emotional effects were excluded from the analyses by controlling for the psychological characteristics of the participants.

Previous findings of SUD studies have been somewhat inconsistent but an association between abnormal gamma activities and the abuse of stimulants has been consistently reported. EEG studies show that individuals who use cocaine exhibit decreased gamma band reactivity, which treatments aim to enhance^51^, and that tobacco use increases gamma activity^52, 53^. However, it remains unclear whether heightened gamma coherence in IGD patients is common in patients with other behavioral addictions, such as GD. Future comparison studies that include patients with IGD and those with other behavioral addictions will help solve this issue. Although the findings of the present study are somewhat limited in that gamma coherence was not correlated with the IAT scores in the IGD group, there was a positive correlation of gamma coherence with the IAT scores in all groups; this may have been due to a ceiling effect of the IAT scores regarding IGD. Thus, to determine whether gamma coherence could be considered as an endophenotype for IGD, future EEG studies investigating the genotypes of IGD patients on a large scale will be necessary.

In the present study, the AUD group tended to show greater coherence in the relatively slow theta band compared to the HCs. Although previous studies have shown that increased theta oscillations may be a neuropathological marker of AUD^54, 55^, the present results suggest that there were no differences in EEG synchrony between AUD patients and HCs when psychological variables were controlled for. This may be due to the suppression mechanism associated with theta coherence that is shared by alcohol and the investigated psychological variables, including depression^56^. The psychological comorbidities that affected the EEG data of the AUD patients in the present study may be explained by the findings of a previous study^26^. We conducted further analyses to clarify this issue. As a result, all groups exhibited positive correlations between theta coherence and depression and between theta coherence and impulsivity. More specifically, scores on the BDI-II were positively correlated with right fronto-central, right fronto-temporal, and right fronto-parietal theta coherence values (*P-*values < 0.010) while scores on the BIS-11 were positively correlated with right fronto-parietal and right centro-parietal theta coherence values (*P-*values < 0.010). Taken together, these findings indicate that AUD patients showed increased theta coherence and that this feature may be more sensitive to psychological comorbidities in AUD patients than in IGD patients. There were no significant pure addictive effects of AUD that could distinguish it from other populations independently of the effects of the psychological variables.

These findings may also be attributable to the heterogeneity of the AUD patient population. A recent study argued that AUD is a heterogeneous disorder because it is associated with a number of sub-phenotypes that each have a unique profile of drinking patterns, motivations for drinking, and neurobiological underpinnings^57^. Additionally, the relative youth of the AUD patients in the present study may have also influenced the results. Age is an important factor when determining structural brain damage in AUD patients and most previous studies investigating this population have assessed middle-aged participants (see Bühler and Mann^58^ for a review). Because the mean age of the AUD patients in the present study was younger than 30 years, the AUD group may have not included a sufficient number of chronic patients that engaged in long-term alcohol consumption. The present study is also limited in that all participants were asked to abstain from alcohol use for at least 2 weeks prior to participation in this study whereas playing Internet games and Internet usage were not restricted. It is possible that the brain dysfunction of the AUD group was underestimated due to this abstinence but it was impossible to avoid the acute and direct effects of alcohol consumption on brain activity and blood pressure, among other factors. Of these possible explanations, the influence of the psychological variables seems to be the most plausible because the AUD patients tended to show increased theta coherence prior to controlling for these characteristics.

Some researchers have argued that the frontal lobe synchronization of neural resources significantly contributes to correlations between measures of EEG and intelligence^59^. Thus, to rule out the influence of intelligence, individuals with an IQ lower than 80 were excluded from the present study. Additionally, the present study only included psychotropic medication-naïve male participants to control for various other factors that may affect neural signaling^13^. As a result, the present findings cannot be generalized to other populations (females, patients on medication, etc.).

In summary, the present study identified abnormal phase synchrony patterns in patients with IGD and AUD relative to HCs using analyses of resting-state EEG. These results demonstrated that the IGD group had significantly greater high-frequency coherence, particularly for gamma, compared to the AUD patients and HCs. Additionally, the AUD group exhibited tendency of increased coherence in the slow-frequency theta band. The increased gamma coherence of the IGD patients appeared to be independent of the effects of the psychological comorbidities and the gamma coherence of the three groups positively predicted the degree of Internet addiction tendency. Moreover, the heightened tendency of theta coherence in the AUD patients relative to the HCs was influenced by their clinical and psychological status. These findings imply that IGD and AUD are associated with different neural activity patterns and that heightened phasic synchrony in the relatively fast-frequency bands of IGD patients during a resting state may be a useful indicator to distinguish IGD from AUD. Furthermore, the heightened phasic synchrony in the gamma band during the resting state may be an important neurophysiological marker of IGD. The present findings will help to broaden the current understanding of the neurophysiological mechanisms underlying the manifestation of IGD.

## Materials and Methods

### Participants

The present study was conducted in accordance with the Declaration of Helsinki and approved by the Institutional Review Board of SMG-SNU Boramae Medical Center, Republic of Korea. All participants provided written informed consent prior to enrollment. This study included 96 male participants between 18 and 60 years of age who were recruited from the SMG-SNU Boramae Medical Center and the surrounding community of Seoul, South Korea. None of the participants had a history of intellectual disabilities (IQ score ≥ 80), psychotic disorders, or neurological disorders, all were psychotropic medication-naïve, and all were right-handed. Individuals who met the inclusion criteria were assigned to the appropriate group: IGD, AUD, or HC.

IGD was diagnosed based on the criteria of the DSM-5 and individuals who spent more than 4 h per day and/or 30 h per week playing Internet games were also included in the IGD group. The IAT was used to assess the severity of IGD; the average (±standard deviation [S.D.]) IAT score of the IGD patients was 69.267 ± 14.776 and the average times spent playing Internet games each weekday and weekend day were 6.207 ± 3.476 h and 8.172 ± 3.389 h, respectively. Patients in the IGD group consumed fewer than 14 alcohol drinks per week and none met the criteria for both AUD and IGD.

AUD was diagnosed based on the criteria of the DSM-5 and inclusion in this group was restricted to those who consumed at least 7 alcohol drinks per day. The severity of AUD was estimated using the AUDIT; the M ± S.D. AUDIT score of the AUD patients was 24.071 ± 5.422 and the M number of standard alcoholic drinks consumed by this group per day was 11.654 ± 7.397. The AUD patients in the present study played Internet games less than 2 h per day and had abstained from alcohol use for at least 2 weeks prior to participation in the study; abstinence from alcohol was verified by self-report and reports from caregivers.

HCs were recruited from the local community and universities. HC participants played Internet games less than 2 h per day, consumed fewer than 14 alcoholic drinks per week, and did not have a lifetime history of any psychiatric disorder, including IGD and AUD. One participant was excluded due to an IQ score <80 and the data of three participants were not included in the final analyses because their EEG recordings were disrupted by excessive movement or the poor quality of EEG channels (>100 uV Sq). Thus, the present study included a final sample of 92 male participants categorized into three groups: IGD (n = 30), AUD (n = 30), and HC (n = 32).

### EEG recordings

- **EEG data collection**
  Each EEG recording session ran for 10 min (4 min with eyes closed, 2 min with eyes open, and 4 min with eyes closed) as participants sat on a comfortable chair in a shielded room with dim lights. The participants were instructed to avoid drowsiness and movement during the data acquisition period. EEG activity was recorded from 64 sites using Ag/AgCl electrodes based on the modified International 10–20 system in conjunction with vertical and horizontal electrooculograms (EOGs) and one bipolar reference electrode (mastoid). The EEG channels were acquired and amplified at a sampling rate of 1000 Hz (Scan 4.5, Neuroscan, Compumedics; El Paso, TX, USA) using a 0.1–100 Hz online bandpass filter and a 0.1–50 Hz offline bandpass filter. Electrode impedances were kept below 5 kΩ.
  All EEG data were analyzed with Neuroguide (NG) Deluxe 2.6.1 software (Applied Neuroscience; St. Petersburg, FL, USA). Thatcher *et al*. reported that, compared to an average reference and the Laplacian reference, the linked ears (LE) reference is suitable for coherence analyses^60^. Thus, the following 19-channel montage with LE references was adopted for the data analyses: FP1, FP2, F7, F3, Fz, F4, F8, T3, C3, Cz, C4, T4, T5, P3, Pz, P4, T6, O1, and O2. Data from each participant were averaged across the recording epochs for each electrode and only the eyes-closed condition was included in the analysis. Artifacts, including eye muscle movements, were determined via manual visual detection and the automatic system of the NG Deluxe 2.6.1. The M ± S.D. epoch length for the data included in the analyses was 240.020 ± 108.699 sec (range: 20.780 to 439.590 sec) and the mean split-half reliability and test–retest reliability values of the EEG data were 0.950 ± 0.008 and 0.944 ± 0.040, respectively. Neither the epoch length nor the two reliability scores differed among the IGD, AUD, and HC groups (all *P-*values > 0.0167).
- **Coherence**

Continuous EEG data were converted into the frequency domain using the Fast Fourier Transformation (FFT) with the following parameters: epoch = 2 sec, sample rate = 128 samples/sec (256 digital time points), frequency range = 0.5–40 Hz, and a resolution of 0.5 Hz with a cosine taper window to minimize leakage. Following the FFT, coherence values were calculated using the NG 2.6.1 program. To minimize the effects of windowing in the FFT^61^, an EEG sliding average of the 256-point FFT cross-spectral matrix was computed for each subject. The EEG data were edited by advancing in 64-point steps (75% overlap), recomputing the FFT, and continuing with the 64-point sliding window of the 256-point FFT cross-spectrum for the entire edited EEG record. The M, variance, S.D., sum of squares, and squared sum of the real (cosine) and imaginary (sine) coefficients of the cross-spectral matrix were computed across the sliding average of the edited EEG for all 19 leads for a total number of 81 and 1,539 log-transformed elements for each subject. The EEG values of each participant at each electrode were computed for each of the following frequency bands: delta (1–4 Hz), theta (4–8 Hz), alpha (8–12 Hz), beta (12–25 Hz), and gamma (30–40 Hz). Next, the following equation (1) was used to determine coherence^62, 63^:

1 $$\begin{array}{rcl}{coherence}\,(\,{f}) & = & ({{\rm{\Sigma }}}_{{\rm{N}}}({\rm{a}}({\rm{x}}){\rm{u}}({\rm{y}})+{\rm{b}}({\rm{x}}){\rm{v}}({\rm{y}})){)}^{2}+({{\rm{\Sigma }}}_{{\rm{N}}}({\rm{a}}({\rm{x}}){\rm{v}}({\rm{y}})\\  &  & +\,{\rm{b}}({\rm{x}}){\rm{u}}({\rm{y}})){)}^{2}/{{\rm{\Sigma }}}_{{\rm{N}}}({\rm{a}}({\rm{x}}{)}^{2}+{\rm{b}}({\rm{x}}{)}^{2}){{\rm{\Sigma }}}_{{\rm{N}}}({\rm{u}}({\rm{y}}{)}^{2}+{\rm{v}}({\rm{y}}{)}^{2})\end{array}$$

anda(x) = cosine coefficent for the frequency (*f* ) for channel x b(x) = sine coefficent for the frequency (*f* ) for channel x u(y) = cosine coefficent for the frequency (*f* ) for channel y v(y) = sine coefficent for the frequency (*f* ) for channel y ﻿

More specifically, intrahemispheric coherence was examined using the F3-C3, F3-T3, F3-P3, C3-T3, C3-P3, and T3-P3 electrode pairs on the left hemisphere and the F4-C4, F4-T4, F4-P4, C4-T4, C4-P4, and T4-P4 electrode pairs on the right hemisphere. Interhemispheric coherence was calculated between electrode pairs F3-F4, C3-C4, T3-T4, and P3-P4^21^.

### Psychological assessments

- **Wechsler Adult Intelligence Scale**

To calculate IQ, performance on the Wechsler Adult Intelligence Scale (WAIS) was assessed^64–66^.

- **Questionnaires**

***Young’s IAT***

The IAT was used to assess the severity of IGD symptoms^67, 68^. The items on this measure are rated on a 5-point scale ranging from 1 (very rarely) to 5 (very frequently). In the present study, IAT scores were calculated based on the total score for all 20 items and ranged from 20–100.

***AUDIT***

The AUDIT, which was developed by the World Health Organization (WHO), was used to assess problematic drinking behavior. Scores on the AUDIT range from 0 to 4^69^.

***BDI-II***

The ***BDI***-II is a 21-item self-report questionnaire in which each item consists of four statements that reflect the levels of severity for a particular symptom experienced during the past week^70^. Scores for all 21 items are summed to yield a single depression score.

***BAI***

The BAI ***employs*** a 4-point scale to rate the severity of 21 anxiety symptoms experienced during the past week^71^. Scores for the 21 items are summed to yield a single anxiety score.

***BIS-11***

The BIS-11 assesses impulsivity based on three subscales^72^: cognitive impulsiveness (e.g., “I get easily ***bored*** when solving thought problems”), motor impulsiveness (e.g., “I do things without thinking”), and non-planning impulsiveness (e.g., “I am more interested in the present than in the future”).

### Statistical analysis

Analysis of variance (ANOVA) and analysis of covariance (ANCOVA) tests were conducted to assess the demographic and psychological variables. Then, Bonferroni-corrected post hoc comparisons were performed using pairwise analyses; P-values = 0.025 were considered to indicate statistical significance. Prior to the statistical analyses, all EEG variables were transformed using Fisher’s z-transformation to normalize the data distribution. To allow for possible unknown correlations between outcomes that could have arisen from the multiple comparisons, separate generalized estimating equations (GEEs)^73^ were conducted to assess the EEG data from each of the following frequency bands: delta (1–4 Hz), theta (4–8 Hz), alpha (8–12 Hz), beta (12–25 Hz), and gamma (30–40 Hz).

Intra- and interhemispheric coherence levels were analyzed using the following factors: 1) intrahemispheric coherence was evaluated according to group (IGD, AUD, and HC) × region (fronto-central, fronto-temporal, fronto-parietal, centro-temporal, centro-parietal, and temporo-parietal) × hemisphere (left and right) and 2) interhemispheric coherence was evaluated according to group (IGD, AUD, and HC) × region (frontal, central, temporal, and parietal). Next, Bonferroni-corrected post hoc comparisons were conducted using pairwise analyses; P-values = 0.0167 were considered to indicate statistical significance.

The intra-and interhemispheric coherence analyses were conducted in two steps because there were significant or tendency of group differences in the demographic (age, education, and IQ) and psychological (depression, anxiety, and impulsivity) variables (Table 1). First, coherence was analyzed while controlling for the effects of the demographic variables but without controlling for the effects of the psychological variables. Second, coherence was analyzed while controlling for the effects of the demographic and psychological variables to determine the pure effects of addiction (with controls for the psychological variables).

To determine the relationships among the variables, Pearson’s correlation analyses and a regression analysis with 2000 bootstrapped samples were performed; P-values = 0.050 were considered to indicate statistical significance. Bootstrapping is useful for the analysis of neurophysiological data while correcting for multiple comparisons and data distribution^74^. All statistical analyses were performed with SPSS 23.0 software (SPSS Inc., Chicago, IL, USA).
